# Association between metabolic syndrome and prevalent skin diseases: A systematic review and meta‐analysis of case‐control studies

**DOI:** 10.1002/hsr2.1576

**Published:** 2023-09-25

**Authors:** Sogand Sodagar, Yekta Ghane, Amirhossein Heidari, Nazila Heidari, Elaheh Khodadust, Seyyed Amir Yasin Ahmadi, Farnoosh Seirafianpour, Hamid Baradaran, Azadeh Goodarzi

**Affiliations:** ^1^ School of Medicine Iran University of Medical Sciences Tehran Iran; ^2^ School of Medicine Tehran University of Medical Sciences Tehran Iran; ^3^ Faculty of Medicine, Tehran Medical Sciences Islamic Azad University Tehran Iran; ^4^ Preventive Medicine and Public Health Research Center Iran University of Medical Sciences Tehran Iran; ^5^ Razi Drug Research Center Iran University of Medical Sciences (IUMS) Tehran Iran; ^6^ Institute of Endocrinology and Metabolism Iran University of Medical Sciences Tehran Iran; ^7^ Aging Clinical & Experimental Research Team, Institute of Applied Health Sciences University of Aberdeen Aberdeen UK; ^8^ Department of Dermatology Rasool Akram Medical Complex Clinical Research Development Center (RCRDC), School of Medicine, Iran University of Medical Sciences Tehran Iran

**Keywords:** androgenic alopecia, dermatology, hidradenitis suppurativa, insulin resistance, lichen planus, metabolic syndrome, psoriasis, rosacea, seborrheic dermatitis, skin disease, vitiligo

## Abstract

**Background and Aim:**

Metabolic syndrome (MetS) is a well‐known noncommunicable disease that plays a significant role in emerging other chronic disorders and following complications. MetS is also involved in the pathophysiology of numerous dermatological diseases. We aim to evaluate the association of MetS with the most prevalent dermatological diseases.

**Methods:**

A systematic search was carried out on PubMed, Science Direct, Web of Science, Cochrane, as well as the Google Scholar search engine. Only English case‐control studies regarding MetS and any skin disease from the beginning of 2010 up to November 15, 2022, were selected. The study was conducted based on the Preferred Reporting Items for Systematic Reviews and Meta‐Analysis (PRISMA).

**Results:**

A total of 37 studies (13,830 participants) met the inclusion criteria. According to our result, patients with psoriasis, hidradenitis suppurativa (HS), vitiligo, androgenetic alopecia (AGA), and lichen planus (LP) have a higher chance of having MetS compared to the general population. Furthermore, people with seborrheic dermatitis (SED) and rosacea are more prone to insulin resistance, high blood pressure (BP), and higher blood lipids. After pooling data, the meta‐analysis revealed a significant association between MetS and skin diseases (pooled odds ratio [OR]: 3.28, 95% confidence interval: 2.62−4.10). Concerning the type of disease, MetS has been correlated with AGA (OR: 11.86), HS (OR: 4.46), LP (OR: 3.79), and SED (OR: 2.45). Psoriasis also showed a significant association but with high heterogeneity (OR: 2.89). Moreover, skin diseases and MetS are strongly associated in Spain (OR: 5.25) and Thailand (OR: 11.86). Regarding the metaregression model, the effect size was reduced with increasing age (OR: 0.965), while the size increased with AGA (OR: 3.064).

**Conclusions:**

MetS is closely associated with skin complications. Dermatologists and other multidisciplinary teams should be cautious while treating these patients to prevent severe complications resulting from MetS.

## Key points

- Metabolic syndrome (MetS) is one of the major scourges among noncommunicable diseases, with a prevalence of 25%−33% of all individuals suffering from this condition globally.
- Skin manifestations can occur in the case of any pathophysiological disorder that results in a loss of metabolic control or involves inflammatory factors such as interleukins, TNF, and oxidative stress.
- Patients with psoriasis, vitiligo, hidradenitis suppurativa, and lichen planus are more likely to suffer from MetS compared to the general population.
- People suffering from seborrheic dermatitis and rosacea are more prone to insulin resistance, high blood pressure, and higher blood lipids.
- The relationship between discoid lupus, acne, atopic dermatitis, alopecia areata, and blistering diseases like pemphigus vulgaris has not been examined yet in case‐control studies; however, there has been some evidence of relationships in the field of MetS components, including insulin resistance.

## 1 INTRODUCTION

Despite the successful eradication of several old infectious diseases in the world, noncommunicable diseases (NCDs) have become the predominant cause of morbidity and mortality not only for people in the developed world but also for the population of underdeveloped countries.^1^ Metabolic syndrome (MetS) is considered one of the major scourges among all of these NCDs, with a prevalence of 12.5%−31.4% of all individuals suffering from this condition globally.^2^ MetS, also known as “insulin resistance syndrome” or “syndrome X” is a cluster of conditions, including dyslipidemia, hypertension, abdominal obesity, and high blood glucose, that together raise the risk of several serious health problems such as cardiovascular diseases, type 2 diabetes, and even death.^1^, ^3^, ^4^ Diagnosis of MetS is based on different criteria, such as those used by the World Health Organization, National Cholesterol Education Program's Adult Treatment Panel III (NCEP ATP III), and the International Diabetes Federation (IDF), which differ slightly from one another.^1^, ^5^ In the pathophysiology of MetS, insulin resistance is generally claimed to be the most common factor.^5^, ^6^ In detail, the poor insulin response in the target cells, including the muscle, liver, and fat tissue, to uptake the blood glucose leads to borderline glucose levels. In the beginning, the pancreas secrets more insulin to achieve euglycemia. Over time, however, an excessive amount of glucose remains in the blood as the cycle progresses.

Due to the increasing prevalence of MetS as a global health concern, the correlation of MetS with other diseases related to the immune system or inflammation is under investigation.^7^ In the case of any pathophysiological disorder that results in a loss of metabolic control, skin manifestations can occur.^6^ To cite an example, psoriasis, hidradenitis suppurativa (HS), acanthosis nigricans, lichen planus (LP), vitiligo, and atopic dermatitis are known to be associated with MetS.^3^ The accumulation of fat in MetS, along with a gradual increase in insulin resistance, causes a cascade of hormonal changes, such as effects on the growth hormone.^8^ Hormones follow the principle of synergistic self‐regulation. Consequently, skin diseases that are influenced by androgenic hormones, such as acne or androgenic alopecia (AGA), may deteriorate.^7^, ^8^ In addition, there is evidence that inflammatory factors such as IL‐17, IL23, TNF, and oxidative stress play a significant role in many autoimmune and inflammatory skin conditions.^7^ Likewise, these factors are present in the body in greater amounts in patients with MetS; therefore, the pathophysiology of both conditions can be described by the same pathways. In this study, we sought to systematically assess the most prevalent skin diseases with the highest association with MetS.

## 2 MATERIALS AND METHODS

The current systematic review was carried out based on the Preferred Reporting Items for Systematic Reviews and Meta‐Analysis (PRISMA) checklists. The checklists are attached as supplementary documents (Supporting Information: Tables S1 and S2).

### 2.1 Search strategy

A thorough systematic search was performed in four databases, including PubMed, Science Direct, Web of Science, and Cochrane, as well as the Google Scholar search engine. A complete list of search terms (keywords and MeSH terms) is mentioned in supplementary documents (Supporting Information: Table S3).

### 2.2 Eligibility criteria and study selection

In this systematic review, case‐control studies with an available English full text published from the beginning of 2010 up to November 15, 2022, were eligible for inclusion. Our primary outcome based on the eligible source populations in the case group were individuals of any age who were suffering from a skin condition and prone to MetS. Reviews, meta‐analyses, guidelines, case reports, case series, and experimental studies (in vitro/ex vivo or animal studies) were excluded.

### 2.3 Data extraction

Extracted data from the studies are as follows: (I) study characteristics (author, year, country, design, sample size, and type of skin disease), (II) patients' characteristics (mean age, the number of cases in both case and control groups, and gender distribution), and (III) results (prevalence of MetS in both case and control groups). Microsoft Excel software, version 16.64, was used for data extraction.

### 2.4 Risk of bias assessment

Two investigators evaluated the methodological quality of the selected publications and the risk of bias independently. Newcastle−Ottawa scale was utilized for assessing the quality of nonrandomized studies, which were included in this systematic review and meta‐analyses^9^ (Supporting Information: Table S4).

### 2.5 Statistical synthesis and analysis

Stata 16.0 (StataCorp LLC) was used for statistical analyses and graphics production. In each study, odds ratios (ORs) were calculated for both patients with skin disease and the control group, presenting values through forest plot graphs with a 95% confidence interval (95% CI). The effect sizes of the mean difference in age, gender, and prevalence of MetS between patients with skin disease and controls were calculated with the mean ± standard deviation (SD). The *Q*‐statistic assessed the heterogeneity of the included studies. *I* ^2^ identified the extent of true heterogeneity using the formula *I* ^2^ = ([Q−degrees of freedom]/Q × 100%).^10^ Pooled effect sizes were calculated using the DerSimonian−Laird random‐effect model if heterogeneity was high (*I* ^2^ > 50% or *p* ˂ 0.1). In the absence of heterogeneity, a fixed‐effect model was used.^11^ We conducted subgroup and sensitivity analyses as well as meta‐regression to probe the origins of heterogeneity further. A funnel plot was used to detect publication bias.^12^ *p* Values < 0.01 were considered statistically significant.

## 3 RESULTS

### 3.1 Search results

**Figure 1:**
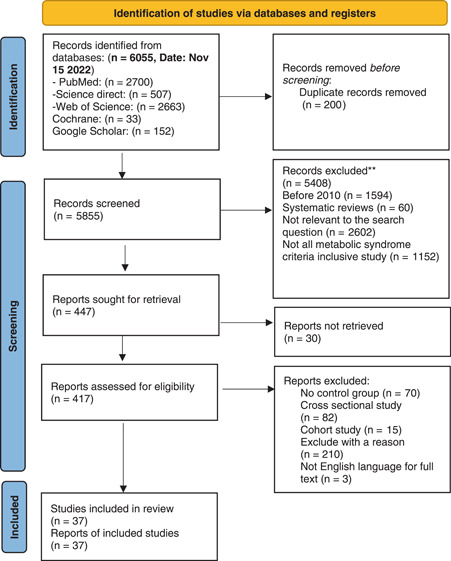
PRISMA 2020 flow diagram for new systematic reviews, which included searches of databases and registers only. PRISMA, preferred reporting items for systematic reviews and meta‐analysis.

A total of 6055 records were detected in the search up to November 15, 2022. The number of 200 duplicates were detected and removed. In the first and second phases of the screening, 5855 studies were reviewed by two independent reviewers who read the titles and abstracts to ensure their quality to be selected. Disagreements between the reviewers were resolved with discussion or the consensus of the corresponding authors. Among these articles, 5408 articles were excluded. In the last screening phase, full texts of 417 articles were reviewed based on the inclusion criteria, and a total of 37 publications were included for data extraction. Figure 1 illustrates the PRISMA flowchart of the current study.

### 3.2 Characteristics of the eligible studies

A total of 37 chosen studies discussed the correlation between MetS and skin diseases, including 21 articles on psoriasis, five on AGA, four on LP, one on HS, two on rosacea, two on vitiligo, and two on seborrheic dermatitis (SED).

### 3.3 Qualitative evaluation of the association between MetS and skin diseases

#### 3.3.1 AGA

Five case‐control studies have been conducted to evaluate the relationship between MetS and AGA with 574 patients in Spain, Thailand, Turkey, and Pakistan. Three studies used the NCEP ATP III criteria for diagnosing MetS, while one study utilized the IDF 2005 diagnostic criteria. In all five studies, MetS prevalence was reported to be significantly higher in patients suffering from AGA.^13^, ^14^, ^15^, ^16^, ^17^ Comprehensive details of the prevalence of MetS and our included skin diseases are illustrated in Table 1

**Table 1:** Characteristics of eligible studies included in our study based on the type of skin disease.

Study (year)	Country	Study design	Type of disease	Disease diagnosis	MetS diagnostic criteria	Number of patients		Age (mean ± SD)		Gender distribution (M: male, F: female)	Result
						Case with	Control without	Case	Control		
Arias‐Santiago et al. (2010)^14^	Spain	Case‐control	AGA	At least type III, according to the Ebling classification	The adult treatment panel‐III	35	35	45.71	48	M: 100%	57.1% of the patients with AGA had MetS compared to 14.3% of the controls (*p* < 0.0001)
Arias‐Santiago et al. (2010)^13^	Spain	Case‐control	AGA	Ebling stage III or above for men, and Ludwig stage II or above for women	The adult treatment panel‐III	77	77	47.1	45.1	M: 51.9% F: 48.1%	60% of male patients with AGA (OR:10.5, 95% CI: 3.3−32.5) had MetS. 48.6% of female patients with AGA (OR: 10.73, 95% CI: 2.7−41.2) had MetS. 12.5% of males and 8.1% of females in control (*p* < 0.0001)
Pengsalae et al. (2013)^15^	Thailand	Case‐control	AGA	No access to the full text	No access to the full text	40	40	‐	‐	M: 100%	Patients with AGA had 3.48 times more risk for MetS compared to the control group (OR: 3.48, 95% CI:1.25−9.75, *p* = 0.015) than the control group (OR: 3, *p* = 0.01
Ertas et al. (2016)^16^	Turkey	Case‐control	AGA	The Hamilton−Norwood and the presence of vertex hair loss	IDF 2005	51	17	31.31	29.41	M: 100%	49.01% of AGA had MetS compared to 11.76% of controls
Sheikh et al. (2021)^17^	Pakistan	Case‐control	AGA	The Hamilton−Norwood scale	The adult treatment panel III	101	101	27.77	27.38	M: 100%	Patients with AGA had an almost fourfold increase in risk for MetS (*p* = 0.016)
Baykal et al. (2015)^18^	Turkey	Case‐control	LP	Confirmed with a skin biopsy	NCEP ATP III (2)	79	79	47.11	46.9	M: 36.7% F: 63.3%	26.6% of LP patients had MetS compared to 12.7% in control (*p* = 0.045). In patients with mucosal involvement, the difference was also higher: (34.5% vs. 8.3%)
Eshkevari et al. (2016)^19^	Iran	Case‐control	LP	Confirmed with skin biopsy in suspected ones	NCEP ATP III	70	70	51.4	48.4	M: 40.8% F: 59.2%	The prevalence of MetS among LP patients was 58.6% in comparison with 17.1% in controls (OR: 6.83; *p* < 0.001).
Kumar et al. (2019)^21^	India	Case‐control	LP	Histopathological findings	NCEP ATP III	75	75	Not mentioned	Not mentioned	Not mentioned	The prevalence of MetS in the case group and control was 34.67% and 14% so that was insignificant (*p* = 0.656)
Kuntoji et al. (2016)^20^	India	Case‐control	LP	Clinical findings and biopsy	NCEP ATP III	50	50	41.2	39.4	M: 57% F: 43%	6% of LP patients had MetS versus 2% in the control group, which was insignificant (*p* = 0.617), Whereas there was a significant association between dyslipidemia and LP, 38% in patients and 6% in controls (*p* < 0.001)
Akin Belli et al. (2016)^22^	Turkey	Case‐control	Rosacea	Based on the National Rosacea Society criteria	IDF 2005 (1)	47	50	50.8	50.9	M: 23.7% F: 76.3%	There was no significant difference between the rosacea and control group for MetS (*p* = 0.186), whereas the rate of insulin resistance was significantly higher in the rosacea group (*p* = 0.009)
Ozbagcivan et al. (2020)^23^	Turkey	Cross‐sectional, case‐control	Rosacea	Based on the National Rosacea Society criteria	Assessed according to the consensus criteria	100	100	46.9	46.9	M: 43% F: 57%	The difference between the prevalence of MetS in rosacea (44%) and control (35%) group was insignificant, (*p* = 0.193). It was significant for dyslipidemia (*p* < 0.001), insulin resistance (*p* = 0.035), hypertension (*p* = 0.009)
Sobhan et al. (2020)^24^	Iran	Case‐control	SD	Clinically and histopathologically	NCEP ATP III	39	39	24.9	25.7	M: 35.8 F: 64.2	No significant difference between the control group (7.7%), & the case (20.5%) (*p* = 0.104) A significant difference between waist circumference (*p* for male = 0.044, *p* for female = 0.035) and systolic BP (*p* = 0.001)
Savaş Erdoğan et al. (2022)^25^	Turkey	Case‐control	SD	No access to the full text	No access to the full text	53	50	Not available full text	Not available full text	Not available full text	The prevalence of MetS in each group was not significantly different, 22.4% and 12%, respectively, in case and control (*p* = 0.155) Whereas fasting blood glucose, triglycerides, HOMA‐IR, and Insulin resistance were significantly higher among case group
Sabat et al. (2012)^26^	Germany	Case‐control	HS	Clinically	NCEP‐ATP III	80	100	40	40.9	M: 45.2% F: 54.8%	MetS was significantly more prevalent in patients (40.0%) versus 13.0% in controls (OR: 4.46; 95% CI: 2.02−9.96, *p* < 0.001)
Ataş et al. (2017)^27^	Turkey	Case‐control	Vitiligo	Not mentioned	ATP III updated by NHLBI	63	65	40.1	40.3	M: 47.6 F: 52.4	The prevalence of MetS among vitiligo patients was 38.1% in comparison with 21.5 in controls (*p* = 0.04) This study also showed that vitiligo activity, affected body surface, vitiligo type, and duration were independent risk factors for MS
Sharma et al. (2017)^28^	India	Case‐control	Vitiligo	Not mentioned	NCEP‐ATP III	100	100	43.5	42.3	M: 65% F: 35%	MetS was significantly more prevalent in the vitiligo group (24%) than in controls (12%) (*p* < 0.05) Also, MS was more prevalent in older vitiligo patients. No association between the severity of vitiligo and MetS was found
Al‐Mutairi et al. (2010)^29^	Kuwait	Case‐control	Psoriasis	Not mentioned	NCEP‐ATP III	1835	1835	52.3	52.7	M: 52.5%	The prevalence of MetSamong patients with mild to moderate in comparison with control was (16% vs. 6.76%, *p* < 0.001) The comparison between severe cases and control was (26.3% vs. 6.7%, *p* < 0.001)
Choi et al. (2010)^30^	Korea	Case‐control	Psoriasis	Histopathological examination	IDF2004	197	401	45	46.8	M: 59.6% F: 40.4%	The prevalence of MetS among psoriasis patients was 17.8 versus 11% in controls. (*p* = 0.021)
Nisa et al. (2010)^31^	India	Case‐control	Psoriasis	Not mentioned	NCEP‐ATP III	150	150	37.4	36.3	M: 69.2% F: 30.8%	The prevalence of MetS was higher in the case (28%) than in controls (6%) (OR: 6.09; *p* < 0.05)
Takahashi et al. (2010)^32^	Japan	Case‐control	Psoriasis	Not mentioned	The Japan Committee of the Diagnostic Criteria of Metabolic Syndrome	151	154	53.1	57.2	M: 71.4% F: 28.6%	The prevalence of MetS in case and control was 25.2% and 16.2%, respectively. (OR: 1.74, 95% CI: 0.99–3.05)
Mebazaa et al. (2011)^33^	Tunis	Case‐control	Psoriasis	Not mentioned	NCEP‐ATP III	164	216	46.2	48.6	M: 47.3% F: 52.7%	The prevalence of MetS in case and control was 35.5% versus 30.8%, which was insignificant. (OR: 1.39, CI: 0.88−2.18; *p* = 0.095)
Damevska et al. (2013)^34^	Republic of Macedonia	Case‐control	Psoriasis	Not mentioned	NCEP‐ATP III	122	122	51.5	51.9	M: 57.4% F: 42.6%	The prevalence of MetS in case and control was 24.6% versus 22.9%, which was insignificant. (OR: 1.095, 95%[CI: 0.607−1.974)
Albareda et al. (2014)^35^	Spain	Case‐control	Psoriasis	Not mentioned	2009 consensus criteria	102	102	49.3	48.7	M: 53.9% F: 46.1%	The prevalence of MetS in case and control was 53% versus 35% (*p* = 0.016)
Hernandez et al. (2014)^36^	Mexico	Case‐control	Psoriasis	Not mentioned	NCEP‐ATP III	103	106	48.3	‐	Not available full text	The prevalence of MetS in case and control was 41.7 versus 20% (OR: 1.73, 95% CI: 1.19‐2.53; *p* < 0.001)
Kokpol et al. (2014)^37^	Thailand	Case‐control	Psoriasis	Not mentioned	The harmonized definition of metabolic syndrome	199	199	50.4	49.9	M: 55.8% F: 48.2%	The prevalence of MetS in the case and control was 49.2% versus 30.6%, which was significantly higher. (OR: 2.25, (*p* < 0.001)
Menegon et al. (2014)^38^	Brazil	Case‐control	Psoriasis	Not mentioned	NCEP‐ATP III	350	346	49.8	48.5	M: 40.6% F: 59.4%	The prevalence of MetS in the case and control were 47.1% and 34.7%, respectively (OR: 1.5; 95% CI: 1.1−2.1, *p* = 0.01). The result changed oppositely when variables like sex and age were adjusted. Waist circumference and Body mass index were the variables that remained significantly higher among patients after adjustment
Prathap et al. (2014)^39^	India	Case‐control	Psoriasis	Not mentioned	The IDF consensus worldwide definition of MS	150	150	48.7	51.4	M: 61.6% F: 38.4%	The prevalence of MetS in the case and control were 29.3% and 18%, respectively. (OR: 1.9, 95% CI: 1.1−3.3, *p* = 0.02)
Itani et al. (2016)^40^	Lebanon	Case‐control	Psoriasis	Not mentioned	NCEP‐ATP III	150	150	42.1	41.9	M: 45% F: 55%	The prevalence of MetS in the case and control was 35.3% and 18%, respectively, (OR: 2.4, *p* < 0.001)
Meziane et al. (2016)^41^	Morocco	Case‐control	Psoriasis	Clinically and histologically	IDF	150	300	40.8	Not mentioned	M: 50.6% F: 49.6%	The prevalence of MetS in case and control was 44.7% and 2.7%, respectively, Which was significant. (OR: 26, 95% CI:12.4−54.3, *p* < 0.001)
Ražnatović Durović et al. (2016)^42^	Montenegro	Case‐control	Psoriasis	Not mentioned	Modified Adult Treatment Panel III (ATP III)]	121	126	50	43.7	M: 44% F: 56%	The prevalence of MetS in the case and control were 48.5% and 20.6%, respectively (OR: 2.99)
Sharma et al. (2016)^43^	India	Case‐control	Psoriasis	Not mentioned	NCEP ATP III criteria modified for South Asians	100	100	43.3	44.9	M: 64.5 F: 35.5%	The prevalence of MetS in the case and control were 38% and 12%, respectively. (OR: 4.49, 95% CI: 2.17−9.29, *p* < 0.001)
Girisha et al. (2017)^44^	India	Case‐control	Psoriasis	Not mentioned	The South Asian modified NCEP ATP	156	156	45.5	45.4	M: 75.6% F: 24.4%	The prevalence of MetS in case and control was 28.8% and 16.7%, respectively, which was significant (*p* = 0.01)S
Salunke et al. (2017)^45^	India	Case‐control	Psoriasis	Not mentioned	Asian Modified National Cholesterol Education Program's Adult Panel III SAM‐NCEP criteria	95	95	36.8	36.3	M: 38.9% F:61.1%	The prevalence of MetS in case and control was 38.9% and 21.05%, respectively, which was significant. (OR: 2.39, 95% CI: 1.26−4.55; *p*‐value = 0.007)
Aounallah et al. (2019)^46^	Morocco, Tunis Algeria	Case‐control	Psoriasis	Not mentioned	NCEP ATP III	671	1241	47.2	47.2	M: 52.7% F: 47.3%	The prevalence of MetS in the case and control were 37.4% and 18.3%, respectively, which was significant. (OR: 2.6, 95% CI: 2.1–3.2; *p*‐value < 0.001)
Mahyoodeen et al. (2019)^47^	South Africa	Cross‐sectional case‐control	Psoriasis	Not mentioned	2009 Harmonized Guidelines	103	98	53.3	47.9	M: 42.6% F: 57.4%	The prevalence of MetS in case and control was 52.4% versus 33.7%, respectively. (*p* = 0.007)
Aalemi et al. (2020)^48^	Afghanistan	Case‐control	Psoriasis	Not mentioned	IDF	113	113	13.9	13.4	M: 48.2% F: 51.8%	The prevalence of MetS in case and control was 13.3% versus 2.3%, respectively, (OR: 5.23; *p* = 0.005)
Ma et al. (2021)^49^	China	Case‐control	Psoriasis	Not mentioned	NCEP ATP III	86	100	49.5	46.1	M: 60.7% F: 39.3%	The prevalence of MetS in case and control was 88.3% and 30%, respectively, which was significant (*p* < 0.0001)

Abbreviations: AGA, androgenic alopecia; BP, blood pressure; CI, confidence interval; HS, hidradenitis suppurativa; IDF, International Diabetes Federation; LP, lichen planus; MetS, metabolic syndrome; NCEP ATP III, National Cholesterol Education Program's Adult Treatment Panel III; OR, odds ratio; SD, seborrheic dematits.

#### 3.3.2 LP

With regard to LP, four studies involving 548 patients were performed in India, Iran, and Turkey, questioning the relationship between MetS and LP. All studies applied the NCEP ATP III criteria for detecting MetS. Studies conducted in Turkey and Iran in 2015 and 2016 reported a higher prevalence rate of MetS in LP patients.^18^, ^19^ Nevertheless, MetS was not meaningfully associated with LP in two other studies carried out in India in 2016 and 2019.^20^, ^21^ Of note, LP and dyslipidemia were found to be strongly correlated in the Indian study, based on investigating 100 patients.^20^

#### 3.3.3 Rosacea

Two case‐control studies in Turkey recruited 293 subjects to peruse MetS and rosacea relevance. Women constituted most of the participants in both studies. Despite the lack of a statistically significant link between MetS and rosacea, insulin resistance, hypertension, and dyslipidemia have been profoundly associated with rosacea in both studies.^22^, ^23^

#### 3.3.4 SED

A total of 181 people were enrolled in two studies, both exploring the association between MetS and SED in Iran and Turkey between 2020 and 2022. The MetS and SED did not appear to be related in either study.^24^, ^25^ Besides, in the study conducted in Iran, waist circumference and systolic BP were significantly higher in people with SED.^24^ Also, in the Turkish survey, blood fat and insulin resistance were considerably higher in people with SED.^25^

#### 3.3.5 HS

Regarding HS, an investigation of 180 patients with an average age of 40 in Germany in 2012 revealed that HS and MetS were strongly correlated.^26^

#### 3.3.6 Vitiligo

In terms of vitiligo, the data gathered from 228 subjects in two studies indicate strong evidence of a significant linkage between vitiligo and MetS.^27^, ^28^ In addition, a 2017 Turkish study discovered that vitiligo activity and the level of body involvement, as well as the type and duration of the disease, were independent risk factors for MetS.^27^

#### 3.3.7 Psoriasis

As depicted in Table 1, 21 studies examined the relationship between MetS and psoriasis with a total population of 11,826 patients. All studies had an average age range between 35 and 53 years old, except one study in which children with an average age of 13 years old were under investigation.^48^ Ninteen of 21 studies recorded that psoriasis patients are greatly more affected by MetS.^29^, ^30^, ^31^, ^32^, ^35^, ^36^, ^37^, ^38^, ^39^, ^40^, ^41^, ^42^, ^43^, ^44^, ^45^, ^46^, ^47^, ^48^, ^49^ Howbeit, statistical analysis results, following the removal of adjusted variables in one study, were altered. It should be noted that in the mentioned study, after implementing the changes described above, a strong correlation between psoriasis and large waist size as well as high body mass index (BMI), was found. The two remaining studies were conducted between 2011 and 2013 in the Republic of Macedonia and Tunisia, involving 624 patients. The MetS and psoriasis were found not to be associated in the two cited papers.^33^, ^34^

### 3.4 Meta‐analysis results: Association of seven skin diseases with MetS and subgroup analyses

#### 3.4.1 Prevalence of MetS in seven skin diseases

**Figure 2:**
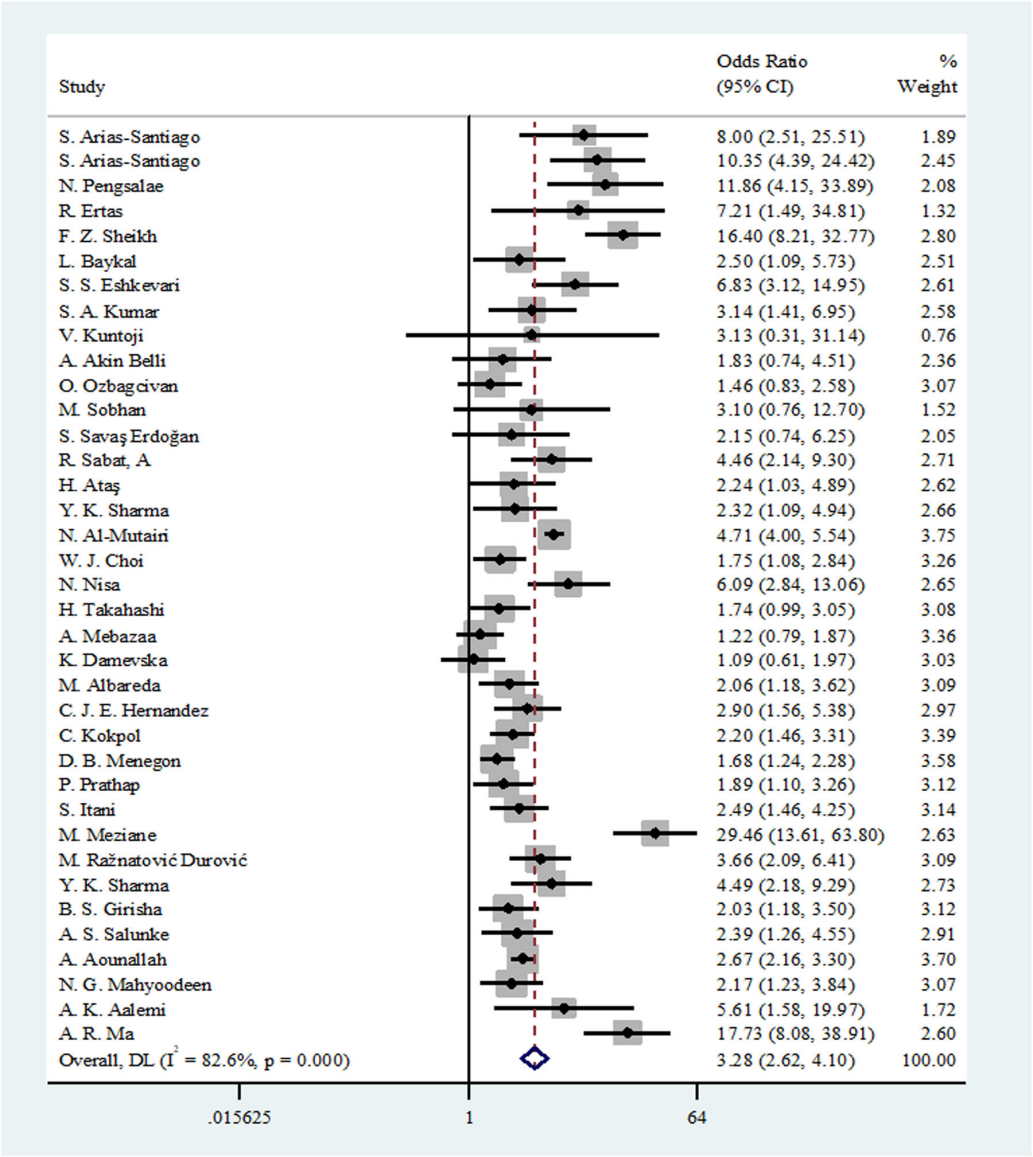
A forest plot of the association between seven skin diseases and metabolic syndrome.

Thirty‐seven studies had adequate data for this meta‐analysis. We used the random effects model due to a high heterogeneity (*I* ^2^ = 82.6%) among the included studies. The pooled OR for the prevalence of MetS between the skin diseases and control group was 3.28 (95% CI: 2.62−4.10). Based on the overall pooled OR, these results suggest that MetS is significantly associated with skin diseases (Figure 2).

#### 3.4.2 Associations of seven skin diseases with MetS: Disease type subgroup analysis

**Figure 3:**
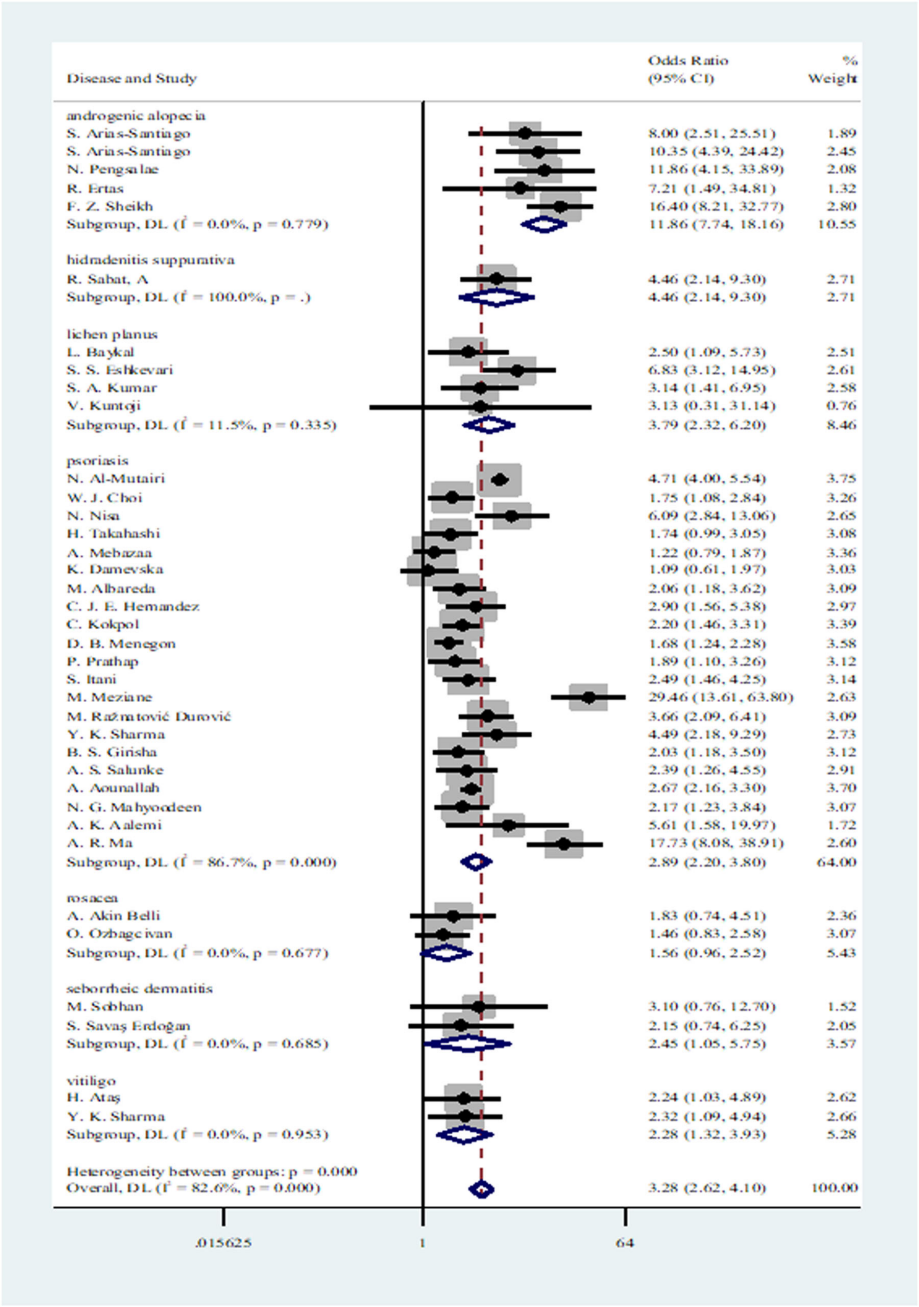
Forest plot showing the association between seven skin diseases and metabolic syndrome based on skin disease type subgroup analysis.

**Figure 4:**
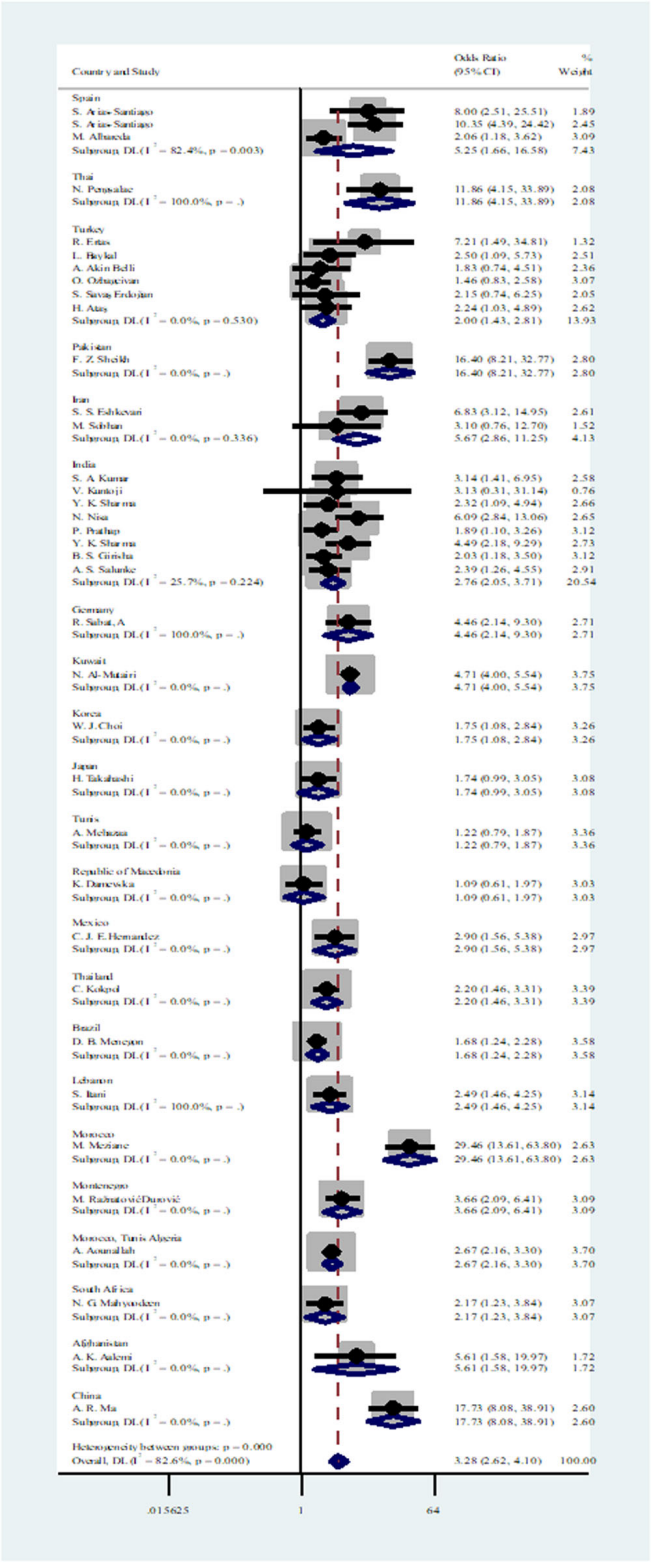
Forest plot showing the association between seven skin diseases and metabolic syndrome based on country subgroup analysis.

In consideration of heterogeneity, further subgroup analyses based on skin disease type and country were conducted (Figure 3 and Figure 4, respectively). Regarding the disease type subgroup, AGA (OR: 11.86, 95% CI: 7.74−18.16), HS (OR: 4.46, 95% CI: 2.14−9.30), and psoriasis (OR: 2.89, 95% CI: 2.20−3.80) demonstrated a significant association with MetS. A moderate association was also found between MetS and LP (OR: 3.79, 95% CI: 2.32−6.20), SED (OR: 2.45, 95% CI: 1.05−5.75), and vitiligo (OR: 2.28, 95% CI: 1.32−3.93). Conversely, rosacea showed a weak association with MetS (OR: 1.56, 95% CI: 0.96−2.52). The MetS appears to be significantly associated with AGA, HS, LP, and SED. Although the studies were highly heterogeneous, psoriasis and MetS were also strongly correlated. In contrast, vitiligo and rosacea appeared to be associated with MetS in a weaker way.

#### 3.4.3 Associations of seven skin diseases with MetS: Country subgroup analysis

In the case of the country subgroup, a meaningful association was found between MetS and skin diseases in Spain (OR: 5.25, 95% CI: 1.66−16.58). In addition, Skin diseases and MetS are strongly associated in Thailand (OR: 11.86, 95% CI: 4.15−33.89). There were no statistical significances found for the OR of Turkey, Pakistan, Iran, India, Germany, Kuwait, Korea, Japan, Tunisia, the Republic of Macedonia, Mexico, Brazil, Lebanon, Morocco, Montenegro, Morocco/Tunis/Algeria, Afghanistan, or China in the analysis.

#### 3.4.4 Meta‐regression analysis results

Lastly, meta‐regression was also performed to explore the potential heterogeneity sources. A meta‐regression was conducted to determine how covariates affected the pooled meta‐analysis result. We included age, year of publication, and sample size as covariates. Furthermore, AG was found to have the greatest impact on the outcome, with a significantly larger effect size than the overall pooled result; a covariate was included in the metaregression to account for this disease.

**Figure 5:**
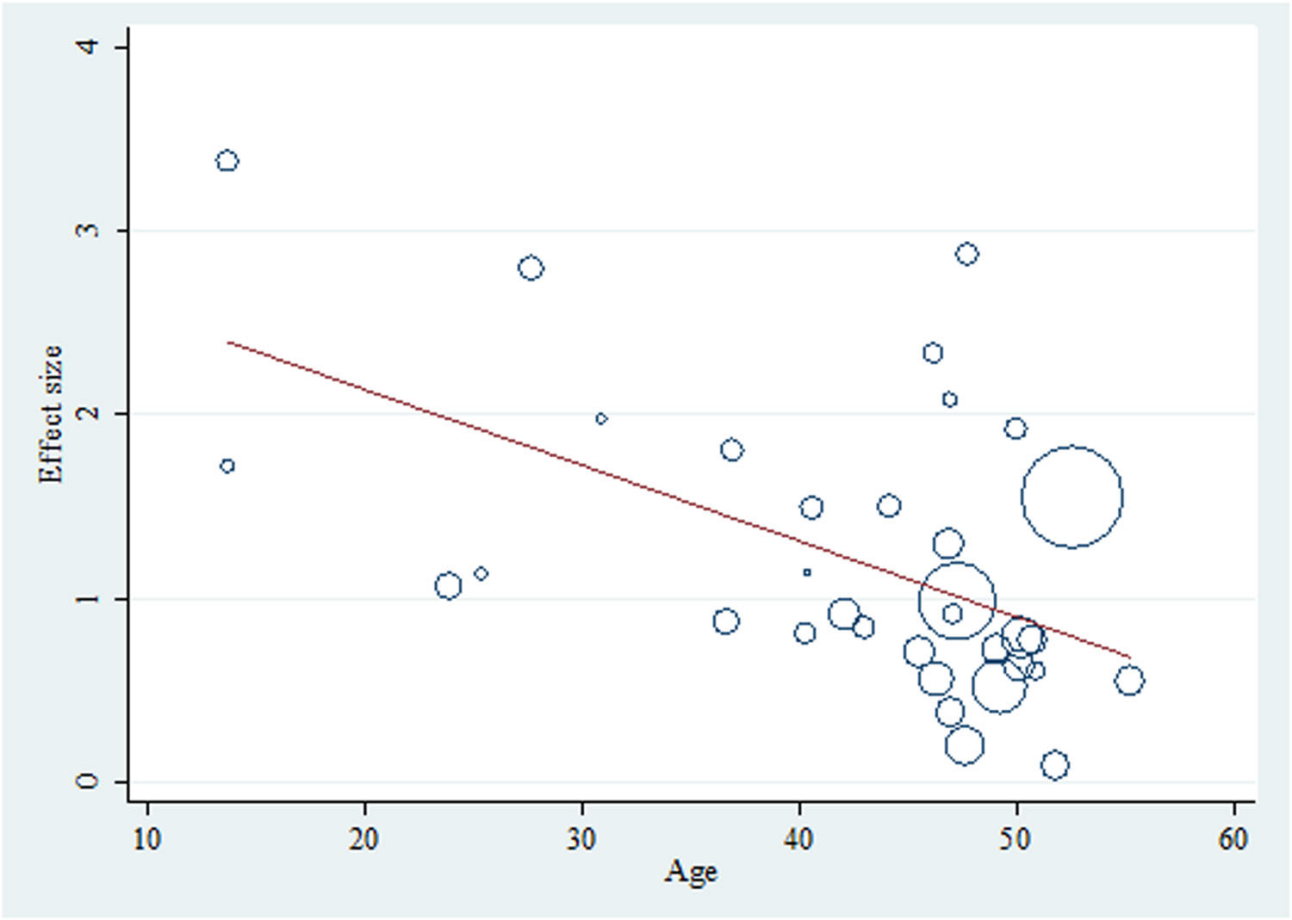
Bubble plot with a fitted meta‐regression line of the effect size and age relationship. Circles are sized according to each estimate's precision (it should be mentioned that the effect size is the logarithmic form of the odds ratio).

**Figure 6:**
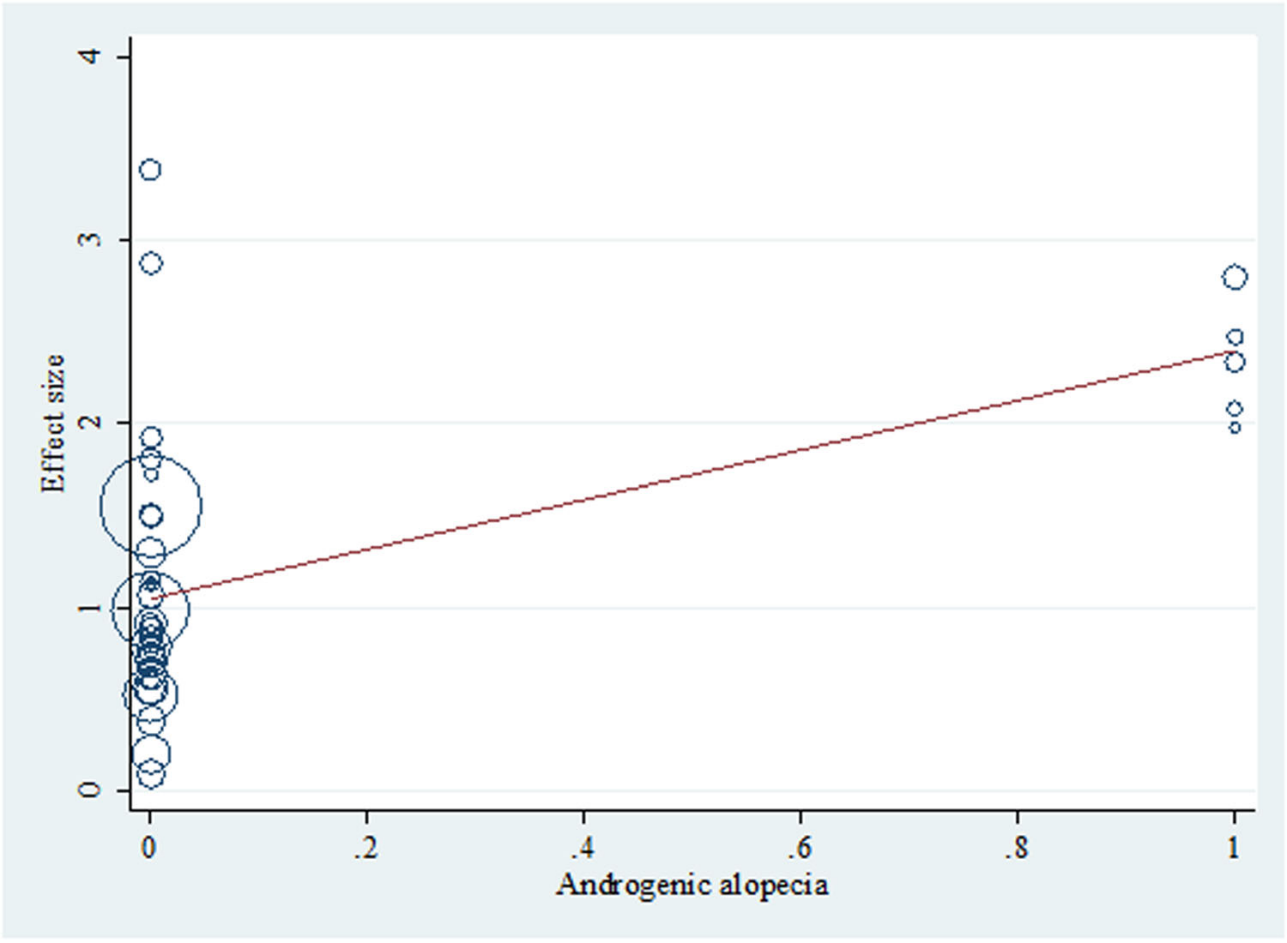
Bubble plot with a fitted meta‐regression line of the effect size and androgenic alopecia relationship. Circles are sized according to each estimate's precision (it should be mentioned that the effect size is the logarithmic form of the odds ratio).

Within utilizing a backward approach, only covariates with *p*‐values <0.1 were retained in the final metaregression model. Among the four variables (age, year of publication, sample size, and AGA), only age and AGA remained in the final model. In summary, each year's increase on average age was associated with a reduction in the effect size (OR: 0.965, Figure 5). In contrast, AGA was associated with increased effect size (OR: 3.064, Figure 6). The crude (unadjusted) results are also presented in Table 2.

**Table 2:** The adjusted and unadjusted (crude model) for final covariates in the metaregression model.

Covariates	Adjusted model			Crude model		
	OR	95% CI	*p* Value*	OR	95% CI	*p* Value
Age	0.965	0.944−0.987	0.003	0.960	0.936−0.983	0.002
Androgenic alopecia	3.064	1.410−6.657	0.006	3.874	1.850−8.107	0.001
Constant	13.572	4.895−37.630	<0.001			

Abbreviations: CI, confidence interval; OR, odds ratio.

* *p* < 0.001 was considered significant.

### 3.5 Sensitivity analyses

Sensitivity analyses can also be carried out on the subgroup results by disease and country. Pooled ORs >1 were found in all subgroups, demonstrating high confidence in the final results.

### 3.6 Publication bias

**Figure 7:**
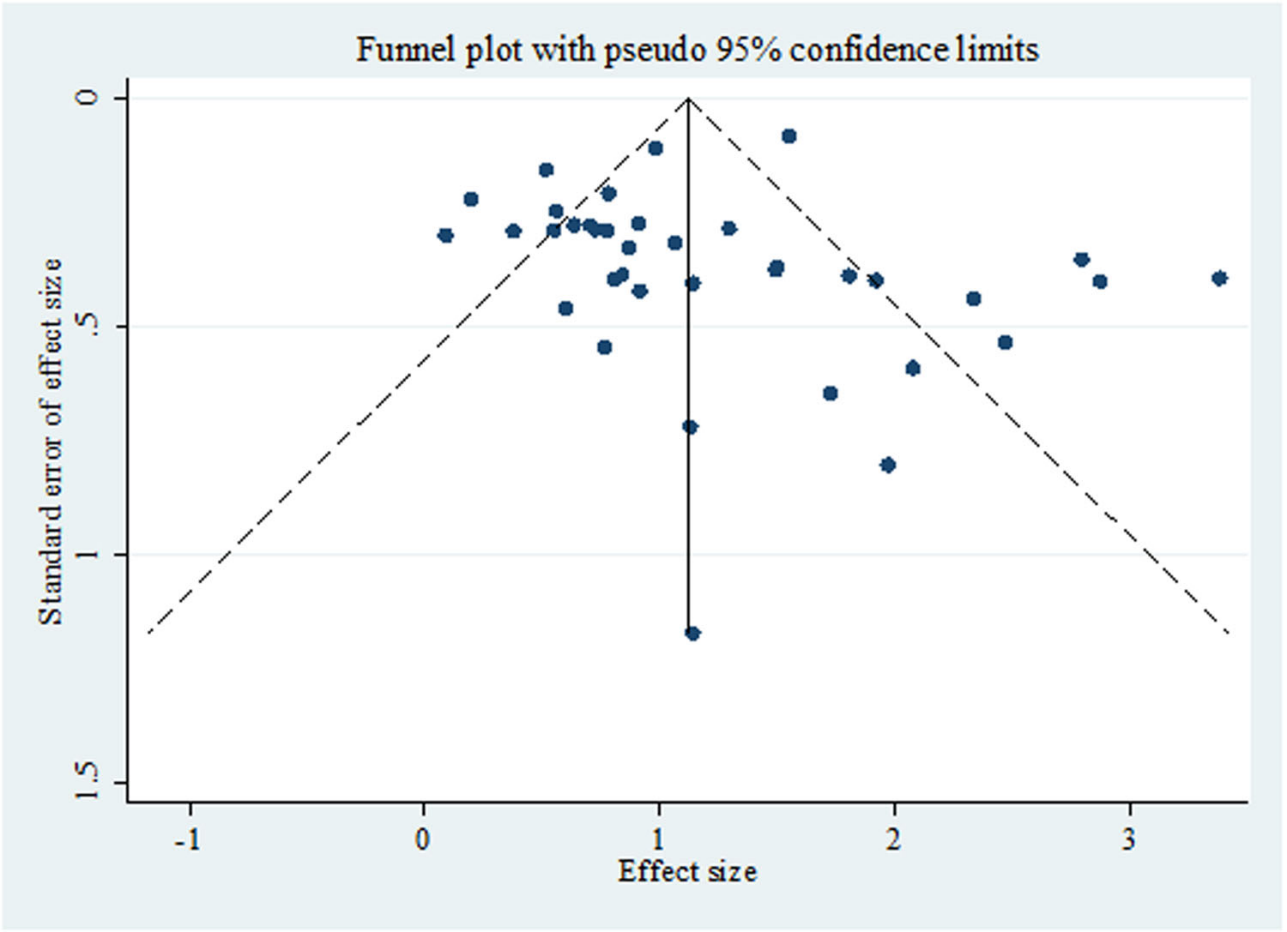
Begg's funnel plot for publication bias (it should be mentioned that the effect size is the logarithmic form of the odds ratio).

This funnel diagram indicates a low probability of publication bias due to the symmetric distribution of studies (Figure 7).

## 4 DISCUSSION

Taking into account the growing prevalence of MetS as a significant health issue worldwide, there has been an increase in the number of studies assessing the relationship between MetS and skin disease in recent years.^7^ MetS is a well‐known NCD identified by its side effects, such as end‐organ damage, elevated levels of glucose and blood lipids, as well as high BP.^50^ This makes patients susceptible to type 2 diabetes and cardiovascular diseases, such as atherosclerosis and hypertension. Insulin resistance is known to be the underlying pathophysiology of MetS.^6^ Evidence of primary and secondary associations between several skin disorders and MetS exists. According to the available findings, we conducted a thorough systematic review of case‐control studies to confirm the validity of this hypothesis. A total of 37 case‐control studies were included; 21 on psoriasis, five articles on AGA, four on LP, one on HS, two on rosacea, two on vitiligo, and two on SED. Regarding conditions such as discoid lupus, types of acne, atopic dermatitis, alopecia areata, and blistering diseases such as pemphigus vulgaris, no case‐control studies have been published to investigate their relationship with MetS. Nonetheless, in a number of publications, the association of the very diseases with the components of MetS, such as insulin resistance, has been discussed.

In our study, the prevalence of MetS was found to be significantly higher in people with AGA. These results are in accordance with a recent systematic review and meta‐analysis conducted by Qiu et al. in which the risk of MetS in patients with AGA was evaluated among 19 studies (pooled OR: 3.46, 95% CI: 2.38−5.05, *p* < 0.01).^51^ Regarding subgroup results, female gender, early onset, and African ethnicity were associated with higher odds of MetS. In addition, individuals with AGA had significantly poorer metabolic profiles, such as BMI, waist circumference, fasting glucose, blood lipids, and BP. In addition, the correlation between LP and MetS has been assessed in our study. According to the results, two studies expounded that the rate of MetS in people with LP was higher than in the normal population^18^, ^19^; however, the same result was not obtained in the other two studies.^20^, ^21^ Based on a recent case‐control study conducted in India, a statistically significant correlation was found between LP and diabetes mellitus, as well as elevated triglyceride levels (TG), increased low‐density lipoprotein (LDL) levels, and low high‐density lipoprotein levels (HDL).^21^ Another case‐control study illustrated an association between dyslipidemia and LP.^20^ Notably, a systematic review and meta‐analysis carried out in 2020 indicated a meaningful correlation between LP and MetS (pooled OR: 2.81, 95% CI: 1.79−4.41, *p* < 0.01).^52^

Considering rosacea, no correlation was found with MetS. Howbeit, the prevalence of insulin resistance, hypertension, and dyslipidemia was remarkably higher in peers suffering from rosacea. Our findings corroborate the results of a systematic review and meta‐analysis with a sample size of 40,752 subjects.^53^ Following this evidence, a meaningful association between high BP (OR: 1.204, 95% CI: 1.09−1.33) as well as insulin resistance (OR: 2.33, 95% CI: 1.18−4.60) and rosacea was depicted, leading to an increased risk of cardiovascular diseases and other related comorbidities. Another investigation illustrated that the level of total cholesterol, LDL, and C‐reactive protein, was higher in patients with rosacea.^54^ These findings support the escalation in the level of inflammation in these patients, which is common in MetS as well. Likewise, the association of rosacea with hypertension and dyslipidemia was evident in the results of the study. Nevertheless, no strong link was found between rosacea and ischemic heart disease. Besides, some evidence indicates a higher level of insulin resistance and obesity in subjects suffering from rosacea.

Moreover, our results suggested that these studies reported no significant association between SED and MetS. Even so, the correlation between this disease and elevated levels of blood lipids, insulin resistance, high BP, and increased waist circumference was found to be meaningful. Previous evidence showed a lower level of HDL in people with SED compared to those without this condition.^55^ Further, a recent cross‐sectional study illustrated that the prevalence of MetS was substantially higher in patients with SED than in the control group (*p* = 0.004).^56^ Furthermore, the number of people with elevated TG levels in the SED group was significantly greater than in the control group (*p* = 0.015). In the patient group, HDL levels were significantly lower in comparison with those in the control group (*p* = 0.050). Both systolic and diastolic BP were also escalated in these patients (*p* = 0.016 and *p* = 0.029, respectively). This study expounded that the presence of SED should be considered a marker for MetS. Thus, further examination is mandatory in this group of patients to confirm the presence of MetS.

Our results also supported the higher prevalence of MetS in HS patients compared to the general population. Based on a subgroup analysis related to the type of HS patient (general patients vs. patients) and age group (adults vs. children), MetS was present in 9.64% of HS patients (OR: 1.82, 95% CI: 1.39−2.25).^57^ An increased risk for MetS was reported in patients of tertiary hospitals and dermatology clinics (OR: 2.82, 95% CI: 0.58−5.06) compared to those treated outside the hospitals (OR: 1.78, 95% CI: 1.34−2.22). A significant correlation was also evident in the included studies considering the pediatric populations (OR: 2.10, 95% CI: 1.58−2.62). In addition, the association between HS and MetS was assessed in an adjusted systematic review and meta‐analysis in 2019.^58^ According to the unadjusted analysis, a significant correlation was found between HS and MetS in adult cases (OR: 1.95, 95% CI: 1.31−2.89, *p* = 0.001). Following adjustment for age, sex, other cardiovascular risk factors, and comorbidities, an adjusted meta‐analysis indicated a significant association (OR: 2.19, 95% CI: 1.70−2.81, *I* ^2^ = 32%, *p* < 0.001). These findings highlight the need to screen patients suffering from HS for MetS.

Furthermore, we found a significant relationship between vitiligo and MetS. In accordance with our results, a recent meta‐analysis study elaborated that vitiligo is associated with diabetes (pooled OR: 3.30, 95% CI: 2.10−5.17) and obesity (pooled OR: 2.08, 95% CI: 1.40−3.11).^59^ As well, the prevalence of hypertension was reported to be 19.0% in vitiligo cases (95% CI: 2.0%−36.0%). However, another recent meta‐analysis proposed that patients suffering from vitiligo did not have a greater risk of developing MetS than control patients (OR: 1.66, 95% CI: 0.83−3.33, *p* = 0.01).^60^ Despite that, there were significant elevations in fasting glycemic index (mean difference 5.35, 95% CI: 2.77−7.93, *p* < 0.001) and diastolic BP (mean difference 1.97, 95% CI: 0.02−3.92, *p* = 0.05) in patients with vitiligo in comparison with control patients. It is important to note that vitiligo patients shall be monitored in terms of the indicators, including BMI, blood sugar, and BP levels.

Despite the heterogenicity of the studies in the case of psoriasis, our results indicated a strong correlation with MetS. In line with our study, a systematic review with a total of 137,053 participants illustrated that psoriasis was correlated with MetS (combined OR: 2.02, 95% CI: 1.67−2.43).^61^ Moreover, another systematic meta‐analysis with the Latin American population claimed that psoriasis and MetS are significantly correlated (pooled OR: 1.66, 95% CI: 1.27−21.42).^62^ Further, a higher risk of MetS was detected in patients with chronic and severe forms of psoriasis (pooled OR: 6.65, 95% CI: 3.32−13.31). On the other hand, based on another systematic review study with evaluations totaling 25,042 patients, the correlation was weak (OR: 1.42, 95% CI: 1.28−1.65).^63^ Also, the risk of MetS was found to be greater in Middle Eastern studies (OR: 1.76, 95% CI: 0.86−2.67) than in European studies (OR: 1.40, 95% CI: 1.25−1.55).^63^ Lastly, monitoring the components of MetS can be advantageous in subjects with psoriasis. MetS, along with the risk of developing its components, will be more likely to affect patients with severe and active forms of psoriasis.

Our results suggest an increased risk of MetS in patients with skin diseases such as psoriasis, vitiligo, HS, LP, SED, and rosacea. Since all the studies included were case‐control studies, causality and generalizability may be limited. Further cohort studies and randomized trials are warranted to better understand the relationship between MetS and these skin diseases. Besides, MetS and associated complications, including type 2 diabetes, hypertension, and cardiovascular diseases, should not be neglected. These conditions negatively impact affected individuals' quality of life and life expectancy. Patients with these metabolic risk factors must be assessed and managed appropriately to prevent or mitigate complications. Additionally, clinicians can improve long‐term outcomes and overall well‐being by addressing modifiable risk factors and implementing preventive measures for individuals who suffer from these skin diseases.

## 5 CONCLUSION

This systematic review consolidates that patients with psoriasis, vitiligo, HS, and LP are more likely to suffer from MetS compared to the general population. Moreover, the prevalence of increased BP, impaired lipid profile, and evidence of insulin resistance is found to be higher in peers with SED and rosacea than in the general population. Howbeit, there is still no robust evidence regarding an increased risk of MetS in these patients. MetS, along with its irreparable consequences, such as type 2 diabetes, high BP, and cardiovascular diseases, have a huge impact on not only the life expectancy of individuals but also the quality of their lives. Hence, early diagnosis and rapid treatment initiation for metabolic disorders in these patients are of great significance. It is imperative to note that maintaining a healthy lifestyle by following a balanced diet and engaging in regular exercise can notably slow or halt the development and progression of such comorbidities.

## 6 LIMITATION AND RECOMMENDATION

The main limitation of this study is the fact that it cannot be determined whether skin diseases are a risk factor for MetS or vice versa. Moreover, the diagnostic criteria of MetS vary among different studies. The presentation of the statistical reports was not homogeneous in the articles as well. Also, the limited number of case‐control studies in some diseases, such as SED, rosacea, and HS, affects the reliability of the results. Further, no case‐control studies were found regarding a number of skin diseases, such as atopic dermatitis, alopecia areata, lupus, and discoid lupus. There has been no case‐control study investigating the relationship between acne and MetS despite numerous studies related to insulin resistance.

## AUTHOR CONTRIBUTIONS

**Sogand Sodagar**: Conceptualization; data curation; writing—original draft; writing—review and editing. **Yekta Ghane**: Data curation; validation; writing—original draft; writing—review and editing. **Amirhossein Heidari**: Data curation; validation; writing—original draft; writing—review and editing. **Nazila Heidari**: Data curation; validation; writing—review and editing. **Elaheh Khodadust**: Validation; writing—review and editing. **Seyyed Amir Yasin Ahmadi**: Formal analysis. **Farnoosh Seirafianpour**: Data curation; validation. **Hamid Baradaran**: Methodology; project administration. **Azadeh Goodarzi**: Conceptualization; methodology; project administration.

## CONFLICT OF INTEREST STATEMENT

The authors declare no conflict of interest.

## ETHICS STATEMENT

Patients or the public were not involved in our research's design, conduct, reporting, or dissemination plans.

## TRANSPARENCY STATEMENT

The corresponding author Azadeh Goodarzi affirms that this manuscript is an honest, accurate, and transparent account of the study being reported; that no important aspects of the study have been omitted; and that any discrepancies from the study as planned (and, if relevant, registered) have been explained.

## Supporting information

Supporting information.

Supporting information.

Supporting information.

Supporting information.

## Data Availability

All data produced in the present study are available upon reasonable request to the authors.
