# Supplementary material for: Association between metabolic syndrome and prevalent skin diseases: A systematic review and meta‐analysis of case‐control studies
Source: Health Sci Rep. 2023 Sep 25;6(9):e1576. doi: 10.1002/hsr2.1576 (PMC10519158; doi:10.1002/hsr2.1576)
Supplement: Supplementary file 3 — Supporting information. [file HSR2-6-e1576-s002.docx]

**Table S3. Full List of Search Terms and Search Strategies.**

| Database or Search Engine | Search Terms | Number of Results |
| --- | --- | --- |
| Google Scholar | (("Metabolic Syndrome "OR "Insulin Resistance "OR "Abdominal obesity metabolic syndrome") AND ("Skin Manifestations "OR "Skin Diseases "OR "Skin Diseases, Vascular "OR "Skin Diseases, Papulosquamous" OR "Skin Diseases, Eczematous "OR "Skin Diseases, Vesiculobullous "OR "Skin Diseases, Metabolic" OR "Skin Diseases, Genetic "OR "Psoriasis "OR "Vitiligo "OR "Lichen Planus "OR "Lichen planus follicularis" OR "Lichen Planus, Familial" OR "Hidradenitis Suppurativa" OR "Hidradenitis suppurativa, familial" OR "Dermatitis, Atopic" OR "Rosacea" OR "Acne, Adult" OR "Eczema")) | 152  (Screening the first 500 results) |
| PubMed | (("Metabolic Syndrome"[Mesh] OR "Insulin Resistance"[Mesh] OR "Abdominal obesity metabolic syndrome" [Supplementary Concept]) AND ("Skin Manifestations"[Mesh] OR "Skin Diseases"[Mesh] OR "Skin Diseases, Vascular"[Mesh] OR "Skin Diseases, Papulosquamous"[Mesh] OR "Skin Diseases, Eczematous"[Mesh] OR "Skin Diseases, Vesiculobullous"[Mesh] OR "Skin Diseases, Metabolic"[Mesh] OR "Skin Diseases, Genetic"[Mesh] OR "Psoriasis"[Mesh] OR "Vitiligo"[Mesh] OR "Lichen Planus"[Mesh] OR "Lichen planus follicularis" [Supplementary Concept] OR "Lichen Planus, Familial" [Supplementary Concept] OR "Hidradenitis Suppurativa"[Mesh] OR "Hidradenitis suppurativa, familial" [Supplementary Concept] OR "Dermatitis, Atopic"[Mesh] OR "Rosacea"[Mesh] OR "Acne, Adult" [Supplementary Concept] OR "Eczema"[Mesh] )) | 2700 |
| Web of Science | (ALL=("Metabolic Syndrome") OR ALL=("Insulin Resistance") OR ALL=("Abdominal obesity metabolic syndrome")) AND (ALL=("Skin Manifestations") OR ALL=("Skin Diseases") OR ALL=("Skin Diseases, Vascular") OR ALL=("Skin Diseases, Papulosquamous") OR ALL=("Skin Diseases, Eczematous") OR ALL=("Skin Diseases, Vesiculobullous") OR ALL=("Skin Diseases, Metabolic") OR ALL=("Skin Diseases, Genetic") OR ALL=("Psoriasis") OR ALL=("Vitiligo") OR ALL=("Lichen Planus") OR ALL=("Lichen planus follicularis") OR ALL=("Lichen Planus, Familial") OR ALL=("Hidradenitis Suppurativa") OR ALL=("Hidradenitis suppurativa, familial") OR ALL=("Dermatitis, Atopic") OR ALL=("Rosacea") OR ALL=("Acne, Adult") OR ALL=("Eczema")) | 2663 |
| Science Direct | ("Metabolic Syndrome" OR "Insulin Resistance" OR "Abdominal obesity metabolic syndrome") AND ("Skin Manifestations" OR "Skin Disease") | 507 |
| Cochrane | ("Metabolic Syndrome" OR "Insulin Resistance" OR "Abdominal obesity metabolic syndrome") AND ("Skin Manifestations" OR "Skin Disease") | 33 |
