# Supplementary material for: Association between metabolic syndrome and prevalent skin diseases: A systematic review and meta‐analysis of case‐control studies
Source: Health Sci Rep. 2023 Sep 25;6(9):e1576. doi: 10.1002/hsr2.1576 (PMC10519158; doi:10.1002/hsr2.1576)
Supplement: Supplementary file 4 — Supporting information. [file HSR2-6-e1576-s001.docx]

**Table S4.** Quality assessment of case-control studies included in this systematic review and meta-analyses.

|  |  | **Risk of bias** | | | | | | | | |
| --- | --- | --- | --- | --- | --- | --- | --- | --- | --- | --- |
|  |  | Selection | | | | Comparability | Exposure | | | Overall |
|  |  | Q1 | Q2 | Q3 | Q4 | Q5 | Q6 | Q7 | Q8 |  |
| **Androgenic Alopecia** | S. Arias-Santiago (2010)  (14) | ***** | **-** | ***** | ***** | ****** | **-** | ***** | ***** | 7 |
|  | S. Arias-Santiago (2010)  (13) | ***** | ***** | ***** | ***** | ****** | ***** | ***** | ***** | 9 |
|  | N. Pengsalae (2013)  (15) | ***** | **-** | ***** | ***** | ***** | **-** | ***** | ***** | 6 |
|  | R. Ertas (2016)  (16) | ***** | **-** | ***** | ***** | ****** | **-** | ***** | ***** | 7 |
|  | F. Z. Sheikh (2021)  (17) | ***** | **-** | ***** | ***** | ***** | **-** | ***** | ***** | 6 |
| **Lichen Planus** | L. Baykal (2015)  (19) | ***** | ***** | ***** | ***** | ****** | ***** | ***** | ***** | 9 |
|  | S. S. Eshkevari (2016)  (18) | ***** | ***** | ***** | ***** | ***** | ***** | ***** | ***** | 8 |
|  | S. A. Kumar (2019)  (21) | ***** | **-** | ***** | ***** | ***** | ***** | ***** | ***** | 6 |
|  | V. Kuntoji (2016)  (20) | ***** | **-** | ***** | **-** | ****** | **-** | ***** | ***** | 5 |
| **Rosacea** | A. Akin Belli (2016)  (22) | ***** | ***** | ***** | ***** | ****** | **-** | ***** | ***** | 8 |
|  | O. Ozbagcivan (2020)  (23) | ***** | **-** | **-** | ***** | ****** | **-** | ***** | ***** | 6 |
| **Seborrheic Dermatitis** | M. Sobhan (2020)  (24) | ***** | **-** | ***** | ***** | ***** | ***** | ***** | ***** | 7 |
|  | S. Savaş Erdoğan (2022)  (25) | ***** | **-** | ***** | ***** | ****** | **-** | ***** | ***** | 6 |
| **Hidradenitis Suppurativa** | R. Sabat, A (2012)  (26) | ***** | ***** | ***** | ***** | ***** | ***** | ***** | ***** | 8 |
| **Vitiligo** | H. Ataş (2017)  (28) | ***** | ***** | ***** | ***** | ***** | ***** | ***** | ***** | 8 |
|  | Y. K. Sharma (2017)  (27) | **-** | ***** | ***** | ***** | ****** | **-** | ***** | ***** | 7 |
| **Psoriasis** | N. Al-Mutairi (2010)  (47) | ***** | ***** | **-** | ***** | ****** | ***** | ***** | ***** | 8 |
|  | W. J. Choi (2010)  (44) | ***** | ***** | ***** | ***** | ****** | ***** | ***** | ***** | 9 |
|  | N. Nisa (2010)  (35) | ***** | ***** | **-** | ***** | ***** | ***** | ***** | ***** | 7 |
|  | H. Takahashi (2010)  (30) | ***** | ***** | ***** | ***** | ****** | **-** | ***** | ***** | 8 |
|  | 1. Mebazaa (2011)   (30) | ***** | ***** | **-** | ***** | ****** | **-** | ***** | ***** | 7 |
|  | K. Damevska (2013)  (49) | ***** | ***** | ***** | ***** | ***** | **-** | ***** | ***** | 7 |
|  | M. Albareda (2014)  (46) | ***** | **-** | ***** | ***** | ***** | **-** | ***** | ***** | 6 |
|  | 1. J. E. Hernandez (2014)   (42) | ***** | **-** | ***** | ***** | ****** | **-** | ***** | ***** | 7 |
|  | 1. Kokpol (2014)   (40) | ***** | ***** | ***** | ***** | ****** | **-** | ***** | ***** | 8 |
|  | 1. B. Menegon (2014)   (37) | ***** | **-** | ***** | ***** | ****** | ***** | ***** | ***** | 8 |
|  | P. Prathap (2014)  (34) | ***** | ***** | ***** | ***** | ****** | **-** | ***** | ***** | 8 |
|  | S. Itani (2016)  (41) | ***** | **-** | ***** | ***** | ****** | ***** | ***** | ***** | 8 |
|  | M. Meziane (2016)  (36) | ***** | ***** | ***** | **-** | ***** | ***** | ***** | ***** | 7 |
|  | M. Ražnatović Durović (2016)  (33) | ***** | ***** | ***** | **-** | ****** | ***** | ***** | ***** | 8 |
|  | Y. K. Sharma (2016)  (31) | ***** | **-** | ***** | **-** | ****** | ***** | ***** | ***** | 7 |
|  | B. S. Girisha (2017)  (43) | ***** | **-** | ***** | **-** | ****** | ***** | ***** | ***** | 7 |
|  | 1. S. Salunke (2017)   (32) | ***** | **-** | ***** | ***** | ****** | ***** | ***** | ***** | 8 |
|  | 1. Aounallah (2019)   (45) | ***** | ***** | ***** | ***** | ****** | ***** | ***** | ***** | 9 |
|  | N. G. Mahyoodeen (2019)  (38) | ***** | ***** | ***** | **-** | ****** | ***** | ***** | ***** | 8 |
|  | 1. K. Aalemi (2020)   (29) | ***** | ***** | ***** | ***** | ****** | ***** | ***** | ***** | 9 |
|  | 1. R. Ma (2021)   (39) | ***** | ***** | ***** | ***** | ****** | ***** | ***** | ***** | 9 |
